# The Effect of Ambient Environmental Conditions on COVID-19 Mortality: A Systematic Review

**DOI:** 10.3390/ijerph18126665

**Published:** 2021-06-21

**Authors:** Karla Romero Starke, René Mauer, Ethel Karskens, Anna Pretzsch, David Reissig, Albert Nienhaus, Anna Lene Seidler, Andreas Seidler

**Affiliations:** 1 Institute and Policlinic of Occupational and Social Medicine (IPAS), Faculty of Medicine Carl Gustav Carus, Technische Universität Dresden, 01307 Dresden, Germany; ethel@civita.org.au (E.K.); anna.pretzsch@mailbox.tu-dresden.de (A.P.); david.reissig@tu-dresden.de (D.R.); Andreas.Seidler@mailbox.tu-dresden.de (A.S.); 2 Institute of Sociology, Faculty of Behavioral and Social Sciences, Chemnitz University of Technology, Thüringer Weg 9, 09126 Chemnitz, Germany; 3 Institute for Medical Informatics and Biometry (IMB), Faculty of Medicine Carl Gustav Carus, Technische Universität, 01307 Dresden, Germany; rene.mauer@tu-dresden.de; 4 Department of Occupational Medicine, Toxic Substances and Health Research, Institution for Statutory Social Accident Insurance and Prevention in the Health Care and Welfare Services (BGW), 22089 Hamburg, Germany; Albert.Nienhaus@bgw-online.de; 5 Competence Centre for Epidemiology and Health Services Research for Healthcare Professionals (CVcare), Institute for Health Service Research in Dermatology and Nursing (IVDP), University Medical Centre Hamburg-Eppendorf (UKE), 20251 Hamburg, Germany; 6 NHMRC Clinical Trials Centre, Faculty of Medicine and Health, University of Sydney, Sydney, NSW 2006, Australia; lene.seidler@sydney.edu.au

**Keywords:** COVID-19, SARS-CoV-2, temperature, humidity, precipitation, seasonality

## Abstract

Weather conditions may have an impact on SARS-CoV-2 virus transmission, as has been shown for seasonal influenza. Virus transmission most likely favors low temperature and low humidity conditions. This systematic review aimed to collect evidence on the impact of temperature and humidity on COVID-19 mortality. This review was registered with PROSPERO (registration no. CRD42020196055). We searched the Pubmed, Embase, and Cochrane COVID-19 databases for observational epidemiological studies. Two independent reviewers screened the title/abstracts and full texts of the studies. Two reviewers also performed data extraction and quality assessment. From 5051 identified studies, 11 were included in the review. Although the results were inconsistent, most studies imply that a decrease in temperature and humidity contributes to an increase in mortality. To establish the association with greater certainty, future studies should consider accurate exposure measurements and important covariates, such as government lockdowns and population density, sufficient lag times, and non-linear associations.

## 1. Introduction

SARS-CoV-2 was first identified in Wuhan, China, in December 2019 [1], and quickly reached pandemic status. To date (May 2021), there have been more than 150 million confirmed cases of COVID-19, the infection caused by the SARS-CoV-2 virus, and more than 3 million COVID-19-related deaths worldwide [2].

Ambient weather conditions, such as temperature and humidity, play a multi-faceted role affecting virus transmission. The major form of transmission of SARS-CoV-2 is through droplets and aerosols containing the virus, released during exhalation, talking, singing, or coughing [3,4]. While larger and denser particles sink to the ground, microdroplets are small enough to remain suspended in the air for a long time (hours to days in still air), depending on their size [5]. Temperature and relative humidity influence the amount of time in which aerosols remain suspended. Larger particles exhaled from the lungs initially have a high-water content, but reduce in size through evaporation once they reach the ambient air. The speed of this shrinkage depends on the ambient temperature and humidity, and the final size of the particle (along with its density) will determine whether it stays in the air for a period of time or whether it falls to the ground [5]. Temperature and humidity may also affect the virus’s viability. For SARS-CoV, it was observed in laboratory conditions that the dried virus retained its viability for days in temperatures between 22 °C and 25 °C and relative humidity between 40 and 50%. However, at higher temperature and higher relative humidity, the viability was lost at a quicker rate [6]. Furthermore, low temperature and humidity of the inhaled air may impair the host airway mucosal surface antiviral defense [7].

The effect of weather variables, such as humidity and temperature, have also been reported for seasonal influenza, indicating a preference of the virus for colder temperatures and lower humidity [8,9]. When looking at worldwide trends, one study indicated that virus transmissibility was highest during the winter (“cold-dry”) for regions where humidity and temperature decrease below thresholds of 18–21 °C and 11 g/kg over the year. In regions where these thresholds are not reached, seasonal influenza transmission peaks in months where precipitation occurs the most (“humid-rain”) [9]. Studies also found a correlation between outdoor temperature and SARS cases, and came to the conclusion that the optimal temperature for transmission was between 16 °C and 28 °C, with humidity of 52% and a wind speed of 2.8 m/s [10,11].

In light of the above observations, it can be surmised that there may also be a dependence of SARS-CoV-2 transmission on weather variables. There have been reviews published regarding the association between weather variables and SARS-CoV-2 transmission [12,13,14]. Yet, these reviews showed contradicting results and they did not assess the quality of the included studies, which would put more weight on the conclusions of studies having a lower risk of bias. Furthermore, the reviews also focused on using COVID-19 infection cases as the outcome. This may be an unreliable outcome measure owing to the changing testing conditions and availability of tests, which have varied throughout the timeline of the pandemic and between countries. The variation in testing will result in inaccurate measures of COVID-19 cases, as reported case numbers will increase or decrease as testing is increased or decreased. Instead, using mortality due to COVID-19 or a form of excess mortality in the population will provide a more objective outcome measure that is not dependent on current testing procedures or availability.

We thus conducted a systematic review in order to evaluate whether weather conditions, namely, temperature, humidity, and wind, were associated with COVID-19 mortality as a proxy for transmission. The results may provide a better predictability of the regional COVID-19 pandemic development.

## 2. Materials and Methods

We searched Pubmed, Embase (Ovid), and the Cochrane COVID-19 Study register on 3 July 2020 and again on 4 January 2021 to find all observational epidemiological research on the effect of temperature and humidity on COVID-19 disease mortality published since January 2020. The search strings comprised keywords for COVID-19 and weather combined with Boolean operators, and they were adapted to each database. The database search strings are included in the online supplement (Table S1). A protocol of the systematic review was registered a priori with the PROSPERO database of systematic reviews (PROSPERO ID: CRD42020196055). This systematic review follows PRISMA reporting guidelines [15] (checklist in Supplementary Material S1).

We conducted the search applying no language or geographical restrictions. We considered studies that had not been peer reviewed and that were available as pre-prints.

### 2.1. Eligibility Criteria

The scope of the review was specified according to the Population, Exposure, Comparison, Outcome, and Study Design (PECOS) scheme, shown in Table 1. We included studies that included both temperature and humidity in their models, and excluded studies only investigating univariate correlations and studies that excluded either temperature or humidity in their multivariate models. We included studies using precipitation if humidity was not investigated. When both humidity and precipitation were studied, we included both parameters. We included studies that investigated mortality specifically due to COVID-19 or studies looking at excess mortality.

We considered only observational studies, such as ecological, case series, cross-sectional, case-control, and cohort studies. Letters to the editor were also examined.

### 2.2. Selection Process

We collected search results in an Endnote library, and removed duplicate listings prior to beginning the study selection process. Two reviewers independently screened the titles and abstracts of the search results, and conflicts were resolved by seeking consensus. If the reviewers still could not agree, a third reviewer made the decision. The full texts of the remaining studies were then screened by two independent reviewers, and again, disagreements were discussed in consensus meetings. We recorded reasons for exclusion during the full-text review.

### 2.3. Data Collection Process

One reviewer extracted the data, and a second reviewer checked the extraction for accuracy. Whenever there was missing or unclear information, we tried to obtain it through personal communication with the authors. We extracted the following for each study: study design, region, population size and cases, assessed time period, exposure measurement and characteristics, outcome source and validation, variables adjusted for in the model, analysis methods, and summary of the quantitative results. Furthermore, funding information and conflict of interest statements were noted. The extracted study results included any measures of association, with corresponding 95% confidence intervals (CI), such as relative risks (RRs), odds ratios (ORs), hazards ratios (HRs), and β values.

### 2.4. Risk of Bias Evaluation

The risk of bias was assessed by two reviewers, applying a risk of bias tool established in previous reviews [16,17,18,19,20], but adapted for our research question (Supplementary Table S2). The risk of bias tool has eight domains: (1) recruitment procedure, (2) exposure assessment, (3) outcome source and validation, (4) confounding, (5) analysis methods, (6) chronology, (7) funding, and (8) conflict of interest, which are described below.

#### 2.4.1. Recruitment Procedure

This domain assesses the potential for selection bias. Studies were evaluated as having a low risk of bias if there were no baseline differences among the study groups, or if adjustment techniques were applied to correct for baseline differences.

#### 2.4.2. Exposure Assessment

This domain was considered as low risk if there was high confidence in the accuracy of the exposure assessment (i.e., data collected from weather stations) and if the exposure measurements were geographically close to the outcome measurements—meaning that they could represent the temperature to which the cases were exposed. If, for instance, the average temperature or humidity readings were taken to represent a whole country, this domain was considered high risk, as regional temperature differences were not considered.

#### 2.4.3. Outcome Source and Validations

Risk of bias was assumed to be low for this domain, if the outcome (COVID-19 deaths) was obtained through objective sources, such as from the World Health Organization (WHO), the John Hopkins University Center for Systems Science and Engineering (CSSE) COVID-19 Dashboard, or from government agencies.

#### 2.4.4. Confounding

If major confounding factors were assessed and accounted for in the analysis, then the risk of bias was assumed to be low for this domain. This varied depending on the population included in each respective study. If more than one population within a country (i.e., different cities or states) was studied, then at least the population density would have to be adjusted for in the model, for the study to be listed as low risk of bias. If more than one country was studied, then at least the population density and a measure of the healthcare system would have to be adjusted for. For instance, we judged the gross domestic product (GDP) as an adequate proxy for the healthcare system in a multi-country study. In addition, if the time span of the study was relatively long (i.e., more than a month), government interventions had to be accounted for in the model for this domain to receive a low risk of bias rating.

#### 2.4.5. Analysis Methods

This domain was rated as low risk of bias if it fulfilled several specifications. The authors should have used adequate statistical models to reduce bias, such as considering the fact that new cases of death are influenced by the amount of older, still infectious cases in the population (autocorrelation). Further, similar variables should not have been reiterated in the model (i.e., mean, low, and high temperatures in the same model. Finally, if more than one population was studied (i.e., different countries), a component in the model should have been included to allow for other differences in the population studied (i.e., random effects).

#### 2.4.6. Chronology

This domain was considered as low risk if the temporal relationship could be established (the exposure precedes the outcome). In our study, this meant that a sufficient lag effect between exposure and outcome should have been taken into account (depending on the population and time period studied, at least 22 days: 6 days mean incubation plus 16 days from start of symptoms to death [21]).

#### 2.4.7. Funding

If the study was funded by non-profit organizations and it was clearly not affected by sponsors, this domain was considered low risk.

#### 2.4.8. Conflict of Interest

If the study authors reported not having a conflict of interest, this domain was considered low risk.

#### 2.4.9. Overall Risk of Bias

Domains 1–6 were considered as major domains, while domains 7 and 8 were mi-nor domains. A study could have an overall low risk of bias (high quality) if all the major domains were low risk. If any of the major domains were rated as high or un-clear, then the study was classified as having an overall high risk. Note that the risk evaluations for domains 7 and 8 have no impact on the overall risk of bias of the study.

### 2.5. Data Synthesis

A narrative analysis of the studies was conducted, based on the characteristics and methods of each study. We planned to conduct a random effects meta-analysis if at least two studies were comparable in terms of outcomes and exposures, but this was not the case.

## 3. Results

**Figure 1:**
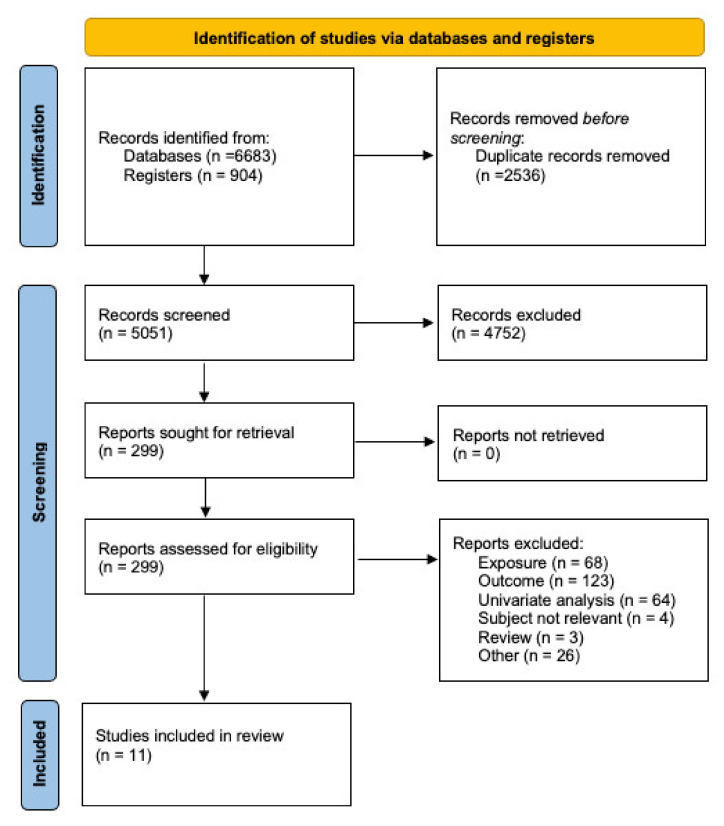
PRISMA flowchart.

Figure 1 shows the PRISMA flowchart of the process followed for the study identification and selection. From 5051 unique identified studies identified (7587 including duplicates), we identified 299 studies for the full text screening. After further screening full texts, eleven studies [22,23,24,25,26,27,28,29,30,31,32] were identified for our review. Both reviewers had a 97% (4923/5051) and 96% (286/299) consensus for the title/abstract and full text screening, respectively. Most of the conflicts between both reviewers were related to the type of analysis—whether it was univariate or multivariate. The most common reasons for exclusion during the full text screening were mortality missing as an outcome, humidity or temperature were missing from the model, or the analysis was only univariate.

### 3.1. Overview of the Studies

All included studies were ecological studies. There were six studies investigating associations worldwide, while two were set in China, one in Pakistan, one in Bangladesh, and one in England. Most studies covered the first quarter or first half of 2020, encompassing primarily the first wave of the pandemic. Most studies investigated the temporal spread of mortality as a function of the weather variables, while two studies instead investigated the spatial spread. Four studies reported relative risks (RRs) and six studies reported β-coefficients based on the results of linear regressions, while one study reported both. Table 2 displays a summary of the study characteristics and results of the studies included in this review. More details of the studies are displayed in Tables S3 and S4.

### 3.2. Quality of the Studies

Detailed information about the quality of each study can be found in Table 3. In general, the quality of the studies was low, mainly due to the “exposure”, “confounding”, “analysis”, and “chronology” domains. Four studies used what we deemed as inaccurate temperature or humidity measurements. For instance, some took average weather conditions to represent a large area (i.e., a country), which may not have characterized the exposure of the cases when infected. Various papers missed what we considered important confounders to include in the model: population density (n = 1); a measure of the healthcare system (n = 1); and government interventions, such as school closures, when the period of the study was prolonged (n = 5). In addition, various studies either did not include an autocorrelation component in their model (n = 6) or it was unclear whether this was done (n = 2). All studies either did not include a lag between the exposure (weather variables) to the outcome (death), or we considered that the lag was insufficient to establish a temporal association. The longest lag considered was 18 days, some four days short of the minimum lag required. Several studies (n = 4) received a high risk of bias in the exposure domain, mostly because the weather measurements were not geographically close to the outcome measurements and, therefore, did not represent the exposure status of the cases. Both studies evaluating the effect of weather variables on mortality by considering the spatial spread of cases were problematic in the exposure and outcome domains. Their exposure assessment was inadequate because the climatic data were either taken from the previous year (2019) or for one day only. Similarly, one study chose one specific day for the outcome assessment, which we evaluated as spurious. The other study used an aggregated three-month mortality rate, which was not precise enough.

Even though no study was found to have a low risk of bias, we considered two studies to be of comparably higher quality: Guo et al. [27] and Fernandez et al. [32], which both used world-wide data. Both of these studies were of high quality in all major domains, except for chronology. If both had used longer lags (they both used 14 days) to account for the time between exposure and outcome, both would have received a high-quality rating.

### 3.3. Effect of Temperature on Mortality

The results on a potential association between temperature and COVID-19 mortality are unclear, yet most studies that did find a consistent association reported a tendency for decreased mortality with increasing temperature. Here, we consider only studies that have provided effect sizes with corresponding 95% confidence intervals, or at least *p*-values. There were three studies (30%) that reported inconsistent associations (both negative and positive associations depending on the analysis) [22,28,30]; one study (10%) with a positive (not-significant) association [30]; five studies (50%) with negative associations [24,25,26,29,31]; and one study (10%) showing small, not significant effects [32]. Ma et al. [22], using non-linear models, reported both negative and positive associations between temperature and mortality depending on the lag days and lag scheme used. Islam et al. [28] reported both positive and negative associations depending on the lag day used when using single day lags, but when using multiple day lags, increasing temperature resulted in increased mortality. Sun et al. [30] had conflicting results (all statistically not significant), depending on the model used. Rehman et al. [26] reported increased mortality with increased temperature, but the effects were not significant. Tzampoglou and Dimitrios [31] found an negative correlation between temperature and mortality, but it was statistically not significant. Su et al. [24] also found decreased mortality with increased temperature. When restricting to countries with over ten days since the first reported case or to countries with over 100 cumulative cases, Wu et al. [25] found a decrease in daily new deaths with increasing temperature (ß = −1.25%; 95% CI: −2.16% to −0.34%). Jiang and Xu [29] also found that daily temperature was negatively correlated to mortality (RR = 0.861; 95% CI: 0.851–0.972). Interestingly, Guo et al. [27] examined non-linear models, which showed that the effect of temperature on deaths was dependent on the temperature range and the lag used. When looking at the single day lags, no statistically significant association was found. When examining the association between COVID-19 mortality and temperature for 14 consecutive days (lag 0–14), mortality at 5 °C was 1.35 times greater than the mortality at 11 °C (RR = 1.35; 95% CI: 1.21–1.51). When the temperature was at 22 °C, the risk in mortality was halved (RR = 0.51; 95% CI: 0.39–0.67). A similar association was found when using a cumulative 7-day lag. Fernandez et al. [32] found only small effects that were not statistically significant.

### 3.4. Effect of Relative Humidity and Precipitation on Mortality

While the studies showed heterogeneous results, they presented a possible negative correlation between mortality and humidity. In this analysis, we include only studies reporting 95% confidence intervals or *p*-values. Ma et al. [22], Rehman et al. [26], and Tzampoglou and Dimitrios [31] showed either no association or the associations were not statistically significant with small effect sizes. Islam et al. [28] reported a positive association for a 0-day lag, but no association for other lag days when using single day lags, but when using multiple day lags, increasing humidity resulted in increased mortality. Fernandez et al. [32], who investigated precipitation instead of relative humidity, showed no correlation between precipitation and mortality.

Although Su et al. [24]’s analyses showed no association between relative humidity and death, the risk of mortality decreased with increasing precipitation (IRR = 0.019; 95% CI: 0.001–0.377). The remaining studies found that an increase in humidity was associated with a decreased risk in mortality. Wu et al. [25] found a −0.46% reduction in daily new deaths associated with a 1% increase in relative humidity (ß = −0.46%; 95% CI: −0.63% to −0.29%), and this effect remained in their sensitivity analyses. Likewise, Sun et al. [30] observed a decreased risk of mortality with increased relative humidity (ß = −4.793; CIs not reported), and the association was statistically significant. Jiang and Xu [29]’s estimations indicated a very small effect of increased relative humidity on the risk of COVID-19 mortality (RR = 0.995; 95% CI: 0.989–1). Guo et al. [27] looked again at non-linear associations, looking at individual lag days and cumulative day lags. While in general, no associations were found in single day lags, associations were found when using cumulative day lags. At a cumulative lag of 0–14 days, the authors found no association of humidity and COVID-19 mortality below 71% relative humidity (RR = 0.98; 95% CI: 0.92–1.05 at 59% relative humidity), but found a statistically significant negative relationship between relative humidity and mortality above 71% relative humidity (RR = 0.86; 95% CI: 0.80–0.92 at 79% relative humidity).

### 3.5. Effect of Wind on Mortality

Three of the included studies investigated the effect of wind on mortality. Su et al. ‘s [24] findings show an increase in mortality with increased wind speed, but the effect was not statistically significant (IRR = 1.155; 95% CI: 0.951–1.403). Rehman et al. [26] found no effect of wind speed on mortality for all study regions. Lastly, Guo et al. [27] explored non-linear associations between wind and mortality. They found an increased risk of mortality with decreased wind speed below 3 m/s (RR = 1.31; 95% CI: 1.16, 1.48 at 2 m/s) and a decreased risk of mortality with increasing wind speeds at wind speeds above 3 m/s (RR = 0.76; 95% CI: 0.70, 0.82 at 4 m/s).

### 3.6. Quantitative Analysis

A quantitative analysis (meta-analysis) could not be conducted as planned for the effect of temperature and wind because of the heterogeneity of the outcome measures and because various studies investigating associations investigated the same population (i.e., worldwide) during the same time frames.

## 4. Discussion

### 4.1. Summary of Results

Our results show some evidence of associations between temperature, humidity, and wind speed on mortality, but they were ambiguous. When associations were found for temperature and mortality, the direction of association indicates a decreased risk in mortality with increasing temperature, supported by 50% of the studies. Half of the studies (five in total) found a decrease in mortality risk with increasing humidity, while others found no association (four studies) or a positive association (one study). Similarly, there were inconsistent findings for the effect of wind on mortality. Considering both higher-quality studies, Fernandez et al. [32], a worldwide study, found no effect of temperature or precipitation on mortality. The other higher-quality study, Guo et al., also a world-wide study that investigated non-linear effects [27], found associations between temperature, humidity, and wind speeds on mortality, depending on the reference point (11 °C, 71% humidity, and wind speed 3 m/s). Overall, if any effect was found, the tendency was for a lower humidity and lower temperature to facilitate virus transmission, indirectly measured by mortality.

The results of our systematic review are in agreement with three previous reviews [12,13,14], suggesting a negative correlation between ambient temperature and humidity and the number of COVID-19 cases, but these previous reviews also report heterogenous findings, with some studies reporting no or even a positive correlation.

### 4.2. Strengths and Limitations of Our Review

A strength of our systematic review is that the title-abstract and full-text screening, the data extraction, and the quality assessment were carried out independently by two reviewers. No language restrictions were applied, and studies only published in preprint severs were included. Further, the study design was published a priori in PROSPERO. Our review only included studies assessing mortality as an outcome. Owing to the variability of testing in the population over time and between countries, using mortality is a more accurate method that is less susceptible to such variations than using COVID-19 cases. The reporting of cases of mortality due to COVID-19 might indeed vary by time and between countries, but we deemed that this bias would be lower than that of reported COVID-19 cases. Mortality is also associated with disease severity, and our results may thus also indicate an association to disease severity. Data on mortality were taken from either official government sources, WHO situation reports, or the John Hopkins University dashboard. These data sources were assumed to be the most reliable, as they are based on local death records. We extracted data on wind if available in the included studies, but unlike temperature and humidity, wind was not a necessary factor for inclusion. Therefore, our results on wind were not representative of all studies investigating wind, but are representative of all studies investigating temperature, humidity, and wind together. A meta-analysis was not possible owing to the heterogeneity of the effect measurements, and because various studies used the same study population during the same time frame. However, we presented the results in a descriptive approach, using the risk of bias evaluation as a supporting point for our conclusions and recommendations.

### 4.3. Risk of Bias of Included Studies and Recommendations for Future Work

The conclusions presented in this systematic review are limited by the high risk of bias of the included studies. No studies were evaluated as having an overall low risk of bias, mostly owing to the exposure, confounders, analysis method, and chronology domains. Most studies missed important confounders, mainly by not including a measure of government interventions in their model. Including factors such as school closures in the model will take the decreased contacts in the population into account, which may have occurred concurrently with temperature changes over time. In addition, some studies missed having a random component in the model to allow for differences in the studied populations. This is especially important when studying different countries, as unmeasured factors such as culture and government type will also have an impact on compliance (or ability to comply) to infection prevention measures. Other studies did not consider autocorrelation, which takes into account that the incident cases on one day are dependent on the number of cases in the past—an important feature of infectious disease epidemiology. Further, no studies considered the necessary lag times to reflect the weather variables at the time of infection. Rather, they tended to use the same time lags as when investigating the association between weather factors and incident cases. Even though most studies only considered linear correlations, it is worth further exploring non-linear associations, as done in Guo et al. [27]. The non-linear associations may be useful to help explain possible contradictory results from linear regressions, or the lack of associations, resulting in heterogeneity. It is possible that the relationships depend on the geographical region studied, with different peaks identified for weather conditions, as for influenza [9]. Studies have shown non-linear temperature and humidity effects on influenza even within one geographical region [33,34,35], and the same may apply for SARS-Cov-2.

All included studies were ecological study designs. However, it may be possible to include epidemiological studies (i.e., cohort and case-control studies) to answer these research questions. With such studies, personal factors, including age and comorbidities, may be included to study the direct effect of weather variables on mortality.

It should be pointed out that some research questions, such as this one, still rely substantially on ecological studies or studies with ecological exposure assessment. Therefore, increased efforts are needed to explicitly address risk of bias assessments to ecological designs as, currently, no standard tools are available for these types of study designs.

### 4.4. Public Policy Implications

In the Northern Hemisphere, which was heavily affected by the COVID-19 pandemic, it is widely expected that a reduction in COVID-19-related disease incidence will occur with rising temperatures in the upcoming summer of 2021. Based on our systematic review, this expectation should be met with caution. Particularly in view of the “action fatigue” observed in many societies, it seems important to consistently continue effective public health measures to contain the spread of the virus until vaccination leads to herd immunity.

## 5. Conclusions

This review shows that some studies appear to confirm the hypothesis that lower temperature and humidity contribute to an increase in cases, although this relationship was not found for all studies. Future studies should principally consider accurate exposure measurements, confounders such as government lockdowns and population density, long-enough lag times, and non-linear associations in order to derive a solid conclusion. Further, because of the lack of unequivocal results regarding the association between temperature and humidity with COVID-19 cases, continued effective public health measures should be implemented despite rising temperatures with seasonal changes, particularly in the upcoming Northern Hemisphere summer in 2021. Preventive methods include contact restrictions, the use of masks, widespread testing, and vaccination.

## Figures and Tables

**Table 1:** Eligibility criteria according to population, exposure, comparison, outcome of interest, and study design.

	Inclusion Criteria	Exclusion Criteria
**P**opulation	General human populations (both sexes, all ages)	All others
**E**xposure(s)	Temperature, humidity *, wind	All other exposures
**C**omparator/control	Not applicable	Not applicable
**O**utcomes	Mortality due to COVID-19 or excess mortality compared to a previous time frame	Other outcomes
**S**tudy design **	Ecological studies, case series, cross-sectional, case-control, and cohort studies	RCTs, qualitative studies, ecological studies, case reports, experiments

* Precipitation may be replaced by humidity; ** congress abstracts, posters, and reviews were excluded.

**Table 2:** Characteristics of included studies.

Author, Year Reference	Study Design	Study Area and Climatic Zone	Time Period of Study	Exposures and Source of Data	Outcome Definition and Source of Data	Confounders/Covariates	Analysis, Lags, and Results
Ma, 2020 [22]	Ecological study	Study area: Wuhan, China Climatic zone: Humid subtropical climate	20 January–29 February 2020	Exposures: Daily average temperature, diurnal temperature range (DTR), and relative humidity Source of data: Shanghai Meteorological Bureau and Data Center of Ministry of Ecology and Environment of the People’s Republic of China	Outcome; COVID-19 deaths Source of data: Official website of Health Commission of Hubei Province	Air pollutants, date of the week, time trends	Analysis: Generalized additive model (GAM) to analyze associations, with a quasi-Poisson link function. Used smoothed spline functions of times to accommodate nonlinear and nonmonotonic patterns between mortality and time. Lag: Examined single day lag and multiple-day average lag effects (0–5 lag) of weather conditions Results: % change of COVID-19 mortality (based on Figures 2 and 3 of the text in Ma et al. 2020 [22])—no quantitative figures could be obtained.
Sobral, 2020 [23]	Ecological study	Study area: World (249 countries)	1 December 2019–30 March 2020	Exposures: Average temperature, maximum temperature, minimum temperature, and precipitation Source of data: National Oceanic and Atmospheric Administration (NOAA) database	Outcome: Daily death rates Source of data: World Health Organization reports	Population density, dummy month (specific month effects), country’s time of exposure to the epidemic (temporal distance, in days, between the first case registered in the territory and the time of study)	Analysis: Multivariate linear regression Lag: No lag effect included Results: Model 1 (average temperature only): ß = 0.053 (*p* < 0.01) Model 2 (average temperature, maximum temperature, minimum temperature, precipitation, exposure time): Death: Average temperature: ß = −0.10 Maximum temperature: ß = 0.01 Minimum temperature: ß = 0.01 Precipitation: ß = 0.34 Model 3 (average temperature, maximum temperature, minimum temperature, precipitation, exposure time, population density, dummy month): Death: Average temperature: ß = −0.10 Maximum temperature: ß = 0.02 Minimum temperature: ß = 0.001
Su, 2020 [24]	Ecological study	Study area: 178 countries/regions (excluding countries/region without COVID-19 cases and some unmatched countries/region (i.e., Taiwan))	22 January–6 April 2020	Exposures: Mean temperature, relative humidity, and precipitation Source of data: Global Surface Summary of the 183 Day (GSOD) via The Integrated Surface Hourly (ISH) dataset (includes global data obtained from the USAF Climatology Center	Outcome: Cumulative mortality rate (CMR) Source of data: John Hopkins University dashboard from Center for Systems Science and Engineering	World Development Indicators dataset (World Bank), urban development (% urban population, population growth, population density), GDP per capita, health, infrastructure (railways, passengers carried), poverty (poverty headcount ratio), science and technology (researchers in R&D) , social protection and labor (cover of social insurance programs, unemployment), mean wind speed	Analysis: Negative binomial regression Lag: No consideration of time (no lag) Results: Cumulative mortality rate Mean temperature (°C): IRR = 0.975 (95% CI 0.887–1.071) Relative humidity (%): IRR = 1.025 (95% CI 0.995–1.056) Mean wind speed (.1 knots): IRR = 1.155 (95% CI 0.951–1.403) Precipitation (0.01 inches) IRR = 0.019 (95% CI 0.001–0.377)
Wu, 2020 [25]	Ecological study	Study area: Worldwide (166 countries excluding China)	December–27 March 2020	Exposures: Temperature and relative humidity Source of data: National Oceanic and Atmospheric Administration Center	Outcome: Daily new deaths Source of data: WHO daily situation reports	Wind speed, median age of national population, Global Health Security Index, Human Development Index, population density, controlling for countries, date of the week and date of the observation to control time trend and cycle	Analysis: Log-linear generalized additive model (GAM) Lag: Single lag days (lag 0, 1, 2, 3). Cumulative effects of average exposure over multiple days assessed using additional analyses (lag 01, 02, 03) Results: Changes in daily new deaths (% change) associated with each 1-unit increase: Temperature (°C): ß = −0.65% (95% CI −1.40% to 0.099%) Relative humidity (%) ß = −0.46% (95% CI −0.63% to −0.29%) Sensitivity analyses: Over 10 days since the first reported case: Temperature (°C): ß = −1.22% (95% CI −2.00% to −0.45%) Relative humidity (%) ß = −0.51% (95% CI -0.68% to −0.34%) Over 100 cumulative cases: Temperature (°C): ß = −1.25% (95% CI −2.16% to −0.34%) Relative humidity (%) ß = −0.53% (95% CI −0.73% to −0.33%)
Rehman, 2020 [26]	Ecological study	Study area: Provinces of Pakistan Climatic zone: Lies in temperate zone with wide variations depending on location	10 March–10 July 2020	Exposures: Daily mean humidity and wind, daily and minimum temperature Source of data: Pakistan Meteorological Department (http://www.pmd.gov.pk/en/), https://www.timeanddate.com/weather/pakistan, https://www.accuweather.com,	Outcome: COVID-19 deaths Source of data: Government of Pakistan http://covid.gov.pk/stats/pakistan and Worldometer Coronavirus cases https://www.worldometers.info/coronavirus/country/pakistan/	Sun status	Analysis: Negative binomial log linear mixed model Lag: No lag Results: Due to lack of space, results summarized in Table S3
Guo, 2020 [27]	Ecological study	415 sites comprising 235 cities from 10 countries and 180 countries	23 January–13 April 2020	Hourly meteorological data (temperature, relative humidity, wind speed) aggregated as daily average meteorological data. Ground-based monitoring network of the World Meteorological Organization global telecommunications system	COVID-19 mortality Johns Hopkins University Center for Systems Science and Engineering (JHU VSSE) The Wind Financial databases (WFD) for detailed information on COVID-19 at city/stae level in Australia, Canada, USA, China, Germany, Italy, Japan, Korea, Norway, and Spain	Date of first reported cases, population density, median age, Global Health Security Index (GHSI), latitude, longitude, intervention policies implemented	Analysis: Negative binomial log linear mixed model Results: Lag 0–14 days Temperature (Reference = 11 °C) 5 °C: RR 1.35 (95% CI: 1.21, 1.51) 22^o^C: RR = 0.51 (95%CI: 0.39, 0.67) Relative humidity (Reference = 71%) 59%: RR = 0.98 (95% CI: 0.92–1.05) 79%: RR = 0.86 (95% CI: 0.80–0.92) Wind speed (Reference = 3 m/s) 2 m/s: RR = 1.31 (95% CI: 1.16, 1.48) 4 m/s: RR = 0.76 (95% CI: 0.70, 0.82) Lag 14 days Temperature (Reference = 11 °C) 5 °C: RR 1.02 (95% CI: 0.99, 1.06) 22^o^C: RR = 0.92 (95%CI: 0.84, 1.01) Relative humidity (Reference = 71%) 59%: RR = 1.00 (95% CI: 0.98–1.02) 79%: RR = 1.00 (95% CI: 0.98–1.02) Wind speed (Reference = 3 m/s) 2 m/s: RR = 1.03 (95% CI: 1.00, 1.05) 4 m/s: RR = 0.98 (95% CI: 0.96, 0.99)
Islam, 2020 [28]	Ecological study	Study area: Bangladesh Climatic zone: Humid monsoon sub-tropical climate	8 March–30 April 2020	Exposures: Night relative humidity (NRH), rainfall, diurnal temperature (TDN), mean temperature (MT), mean relative humidity (MRH), and absolute humidity (AH) Source of data: Bangladesh Meteorological Department (BMD) weather stations	Outcome: COVID-19 death cases Source of data: Bangladeshi government site	None besides the weather parameters shown in results (NRH, TDN, MT, MRH, AH)	Analysis: Compound Poisson generalized linear model, along with a Monte-Carlo method and random forest model Lag: Single and multiple day lags Results: no effect numbers (Figures 5 and 6 in the text in Islam et al. 2020 [28] show a depiction)
Jiang and Xu, 2021 [29]	Ecological study	Study area: Wuhan, China Climatic zone: Humid sub-tropical climate	25 Jan–7 April 2020	Exposure: Daily temperature, relative humidity, and diurnal temperature range Source of data: Weather Channel (www.weather.com)	Outcome: COVID-19 deaths Source of data: Health Commission of Hubei China	No further confounders in the analysis model and no government interventions were included because the whole study period was under strict lockdown	Analysis: Poisson generalized linear model Lag: 18 days Results: Daily temperature ß = −0.149 RR = 0.861 (95% CI: 0.851, 0.872) Relative humidity ß = −0.005 RR = 0.995 (95% CI: 0.989, 1) Diurnal temperature range ß = 0.014 RR = 1.014 (95% CI: 1.003, 1.025)
Sun 2020 [30]	Ecological study	Study area: 317 local authority districts (LADs) in England Climatic zone: Temperate climate	March–May 2020	Exposure: 3-month mean monthly relative humidity and monthly air temperature (from 2019) Source of data: Met Office HadUK-Grid, Gridded Climate Observations on a 1 km Grid over the UK	Outcome: Aggregated three-month England-wide COVID-19 mortality rate. Spatial patterns of COVID-19 mortality compared with non-COVID-19 mortality Source of data: Office for National Statistics	First model: sex, ethnicity (percent Asians, percent blacks), percent of households in poverty, unemployment rate, population density, hospital density annual mean PM_2.5_	Analysis: Variable selection: Lasso technique, spatial autoregressive model (MESS-SAR), Eigenvector spatial filtering model (RES-ESF) Lag: No consideration of time (no lag) Results: First model: OLS Model: Humidity: ß = −8.521 (*p* < 0.001) Air temperature: ß = −0.795 MESS-SAR model: Humidity: ß = −3.715 (*p* < 0.01) Air temperature: ß = 1.512 RE-ESF model: Humidity: ß = −4.793 (*p* < 0.001) Air temperature: ß = 3.852
Tzampoglou and Dimitrios, 2020 [31]	Ecological study	Study area: Worldwide 101 countries (countries with Human Development Index (HDI) < 0.7 excluded from analysis)	March–3 May 2020	Exposures: Monthly average atmospheric temperature (°C), monthly average relative humidity (%), and cumulative precipitation (mm) Source of data: Collected from the Copernicus Program database, estimated from climate reanalysis ERA-Interim and ERA5 Spatial analysis tool of the ArcGIS software was employed to derive the spatial average of variables across the entire territory of each country. After spatial averaging, temporal average values were computed for the March 2020 to May 2020 period.	Outcome: Total deaths per million due to COVID-19 Source of data: European Commission (EC), OurWorldInData.org, and COVID-19 Government Response Tracker, Blavatnik School of Government	Cloud cover (CC), population density (PD), median age (MA), stringency index (SI), delay in first case (FC) and stay at-home order measures (SH)	Analysis: Linear model, variable selection: Lasso and forward stepwise Lag: No lag (no consideration of time) Results: Only two models shown, other models in Table S3 Model A Temperature: ß = −108.9 (95% CI: −307.2, 89.4) Relative humidity: ß = 82.2 (95% CI −125.1, 289.5) Precipitation: ß = 13.4 (95% CI −258.8, 285.6) confounders: CC, PD, MA, SI, FC, SH Model B Temperature: ß = −88.9 (95% CI −259.2, 81.5) Relative humidity: ß = 79.1 (95% CI −126.5, 284.8) Precipitation: ß = −17.9 (95% CI −239.6. 203.8) Confounders: PD, MA, SI, FC, SH
Fernández 2021 [32]	Ecological study	Study area: Worldwide 218 countries	21 January–18 May 2020	Exposures: Maximum, miniumum, and average daily temperature and precipitation Source of data: Downloaded from NASA’s Goddard Earth Sciences Data and Information Services Center (GES DISC). Integrated Multi-satellite Retrievals for Global Precipitation Measurement (IMERG), MERRA-2 (a Modern-Era Retrospective analysis for Research and Applications version 2)	Outcome: Daily confirmed deaths and the total amount of confirmed deaths Source of data: Population-level information (per country), reported by WHO	National Biodiversity Index (NBI), population density, days since last case, days since first case reported in country, country income level, government intervention level	Analysis: Generalized linear mixed models Lag: 14 days Results: Results of Bayesian spatio-temporal regression analysis: All countries Precipitation: ß = 0.000 (95% CI: −0.002, 0.001) Maximum temperature: ß = −0.003 (95% CI: −0.010, 0.005)

CI: confidence intervals; IRR: incidence rate ratio; RR = relative risk; NA: not available.

**Table 3:** Risk of bias in the included studies.

Study ID	Major Domains						Minor Domains		OVERALL
	Recruitment Procedure	Exposure Assessment	Outcome Source and Validation	Confounding	Analysis Method	Chronology	Funding	Conflict of Interest	
Ma et al. 2020 [22]	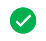	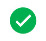	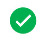	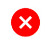	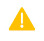	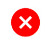	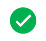	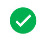	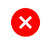
Sobral et al. 2020 [23]	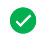	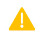	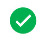	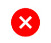	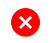	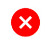	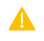	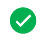	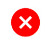
Su et al. 2020 ** [24]	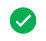	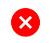	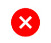	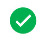	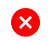	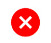	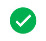	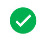	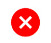
Wu et al. 2020 [25]	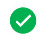	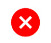	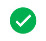	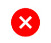	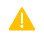	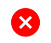	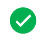	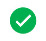	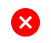
Rehman et al. 2020 [26]	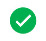	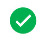	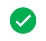	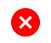	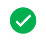	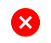	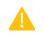	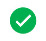	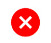
Guo et al. 2020 [27]	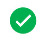	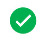	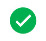	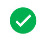	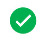	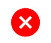	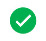	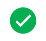	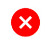
Islam et al. 2020 [28]	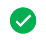	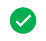	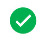	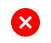	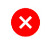	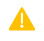	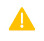	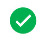	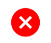
Jiang and Xu et al. 2021 [29]	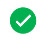	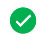	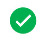	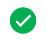	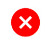	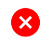	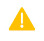	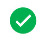	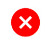
Sun et al. 2020 ** [30]	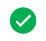	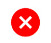	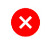	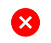	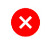	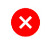	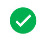	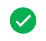	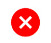
Tzampoglou and Dimitrios et al. 2020 [31]	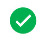	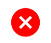	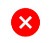	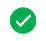	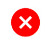	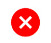	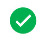	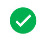	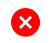
Fernandez et al. 2020 [32]	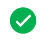	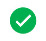	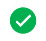	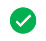	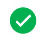	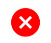	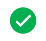	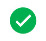	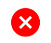

** Spatial correlation 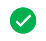: low risk; 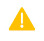: unclear risk; 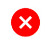: high risk.

## Data Availability

We did not collect own data for this systematic review, and our analyses are based on already published data. Data presented in this study are available in the main text and Supplementary Material (i.e., extraction tables).

## Supplementary Materials

The following are available online at https://www.mdpi.com/article/10.3390/ijerph18126665/s1, Supplementary Material S1: PRISMA 2020 Checklist; Table S1: Search String for Medline (via OVID); Table S2: Risk of Bias Tool; Table S3: Characteristics of Included Studies; Table S4: Results of Included Studies.
