# Supplementary material for: The Effect of Ambient Environmental Conditions on COVID-19 Mortality: A Systematic Review
Source: Int J Environ Res Public Health. 2021 Jun 21;18(12):6665. doi: 10.3390/ijerph18126665 (PMC8296503; doi:10.3390/ijerph18126665)
Supplement: Supplementary file 1 [file ijerph-18-06665-s001.zip › Table S1 S2 S3 S4.pdf]

Table S1. Search String for Databases/Register

MEDLINE (via Ovid)	
1.	(weather or season or temperature or relative humidity or altitude or latitude or environment).tw
2.	(coronavirus or covid* or SARS* or severe acute respiratory or severe acute respiratory syndrome or coronavi* or corona virus).tw.
3.	1 and 2
4.	limit 3 to dd=20200101-20210104
5.	limit 3 to rd=20200101-20210104

Pubmed:

((“weather”[tw] OR “season”[tw] OR “temperature”[tw] OR “relative humidity”[tw] OR “latitude” OR “altitude” OR “environment”) AND (“coronavirus”[tw] OR “covid”[tw] OR “SARS2”[tw] OR “SARS-CoV-2” [tw] OR “severe acute respiratory syndrome”[tw] OR “severe acute respiratory”[tw] OR “coronaviridae”[tw] OR “corona virus”[tw])) AND 2020/01/03:2021/01/04[dp]

Cochrane COVID-19 study register:

Keywords: season, temperature, humidity, altitude, latitude, environment

Table S2. Risk of Bias Tool

Major risk of bias domains	Risk	Criteria	Hints/ notes
**1. Recruitment procedure**<br>***For ecological studies or other analytical studies*†**<br><br>*HINT: We are looking for selection bias: Examples of baseline difference:*<br>• *Protocols for recruitment or inclusion/exclusion criteria were applied differently across study groups*<br>• *Study participants/populations were recruited at different times*<br>• *Study participants/populations were recruited at different times*<br>• *Study participants were recruited from different populations and proportions of participants from each population in each study group are not uniform (consider study base principle)*<br>• *Participation rates were inadequate or not comparable across study groups*	low	☐ There were no baseline differences among study groups or adjustment techniques were used to correct for the differences, OR there is insufficient information about participant selection, but there is indirect evidence that suggests that participant recruitment and inclusion/exclusion criteria was consistent, as described by the criteria for a judgment of low risk of bias.	
	high	There were baseline differences among study groups and no adjustment was used to correct for differences, such as (select all that apply):<br>☐ Protocols for recruitment or inclusion/exclusion criteria were applied differently across study groups.<br>☐ Study participants/populations were recruited at different times.<br>☐ Study participants were recruited from different populations and proportions of participants from each population in each study group are not uniform (study base principle not considered).<br>☐ Participation rates were inadequate or not comparable across study groups.	
	unclear	☐ There is insufficient information about participant selection to permit a judgment.	
**2. Exposure assessment**<br><br>*Example of high risk of bias: average temperature/humidity readings are taken to represent a whole country.*	low	☐ There is high confidence in the accuracy of the exposure assessment# or less established/ less direct exposure measurements are validated against well-established of direct methods† AND<br>☐ Exposure measurements are geographically close to outcome measurements to represent exposure status of the cases.	
	high	☐ Exposure was not accurately measured. #<br>☐ Less established or less direct exposure measurements are not validated and are suspected to introduce bias, which may affect the outcome assessment. †	

Major risk of bias domains	Risk	Criteria	Hints/ notes
		☐ Exposure measurements are not geographically close to outcome measurements and therefore do not represent exposure status of the cases.	
	unclear	☐ Not reported.	
**3. Outcome "deaths/mortality" Source and validation**<br><br>*For COVID-19 deaths, WHO, John Hopkins Dashboard, and government agencies would be considered appropriate sources. There will be populations which may underreport COVID-19 deaths, but it is not possible to quantify or judge the extent of the underreporting. If underreporting is a concern, it should be mentioned in the discussion.*<br><br>*Excess mortality in the populations (i.e., compared to the previous year, will also be accepted as an outcome)*	low	☐ Outcome was objectively ascertained to minimize bias through objective sources (see note).#<br>☐ Measurement methods were similar in the different groups. #	
	high	☐ Outcome was not objectively ascertained. #<br>☐ Measurement methods were different in the groups. #	
	unclear	☐ Not reported.	
**4. Confounding**	low	☐ Major confounding factors were assessed and accounted for.<br>If more than one population within a country was studied: at least population density<br>If more than one country was studied: at least same as above and a measure of the healthcare system was accounted for (i.e., gross domestic product as proxy for differing health system would be acceptable).<br>In addition to above, if time span of study is long (i.e., greater than 1 month): government interventions, such as lockdowns or school closures were accounted for.	Confounders not assessed:
	high	☐ Major confounding factors were not assessed nor were they accounted for.	

Major risk of bias domains	Risk	Criteria	Hints/ notes
	unclear	☐ Not reported.	
**5. Analysis method: methods to reduce research specific bias**<br><br>*Example of similar variables reiterated in different forms: both maximum and minimum temperatures were used in the same model.*<br><br>*It is acceptable when one same variable is used non-linearly (i.e., in quadratic form).*	low	Authors used adequate statistical models to reduce bias:<br>☐ Considered autocorrelation AND<br>☐ Similar variables in different forms were not reiterated in the model (see note) AND<br>☐ If more than one population was studied, a component was included to allow for other differences in the populations studied (i.e., random effects)	
	high	Authors did not use adequate statistical models to reduce bias:<br>☐ Did not consider autocorrelation<br>☐ Similar variables in different forms were reiterated in the model (i.e., both maximum and minimum temperatures used)<br>☐ If more than one population was studied, there was no component included to allow for differences in the populations studied (i.e., random effects)	
	unclear	☐ Not reported.	
**6. Chronology**	low	☐ Temporal relation may be established (exposure precedes the outcome), for example by accounting for a sufficient lag effect between exposure and outcome (i.e., at least 22 days). #	
	high	☐ Temporal relation cannot be established (i.e., no lag effect was included or was insufficient).	
	unclear	☐ Not reported.	

Minor risk of bias domains*	Risk	Criteria	Hints/ notes
**7. Funding**	low	☐ Grant/ non-profit-organizations*<br>☐ Study was clearly not affected by sponsors. *	
	high	☐ Sponsoring organization participated in data analysis.<br>☐ Study was probably affected by sponsors.	
	unclear	☐ Industry, combined industry+grant*, unclear if study was affected by sponsors.<br>☐ Not reported.	

Minor risk of bias domains*	Risk	Criteria	Hints/ notes
**8. Conflict of interest**	low	☐ Reported not having conflict of interest or clear from report/communication that study was not affected by author(s) affiliation.*	
	high	☐ Conflict of interest exists (at least one author).*	
	unclear	☐ Not reported.	

*according to Ijaz et al. (2013), with modifications; # SIGN/CASP

**Overall risk of bias assessment:** General rule for rating domains: if at least one box in domain is marked for "high risk", the domain is high risk.		**Low Risk**	**High Risk**	**Unclear Risk**
Major domains	1. Recruitment procedure			
	2. Exposure assessment			
	3. Outcome source and validation			
	4. Confounding			
	5. Analysis method: methods to reduce research specific bias			
	6. Chronology			
Minor domains	7. Funding			
	8. Conflict of interest			
**General rule for overall rating: Low risk of bias:** low risk in all major domains<br>**High risk of bias:** if not low risk	**Overall assessment:**			

Table S3. Characteristics of included studies

Author, Year<br>Reference number	Study Design	Study area/<br>Population size:<br><br>Number of cases:	Time period of study	Exposures<br>Exposure data source:<br>Exposure characteristics:	Outcome definition:<br>Outcome source of data:
Ma, 2020	Ecological study	Wuhan, China<br><br>2299 COVID-19 deaths in Wuhan (56 deaths/day)	January 20-February 29, 2020	Daily average temperature, diurnal temperature range (DTR), and relative humidity<br><br>Obtained from Shanghai Meteorological Bureau and Data Center of Ministry of Ecology and Environment of the People's Republic of China<br><br>Temperature:<br>Range: 1.8 ^{o}C- 18.7 ^{o}C<br>Mean ± SD: 7.44 ± 3.96 ^{o}C Median: 6.5 ◦C<br><br>DTR:<br>Range: 2 ^{o}C – 17.5 ^{o}C<br>Mean ± SD: 9.15 ± 4.74 ^{o}C<br>Median: 8.70 ^{o}C<br><br>Relative humidity:<br>Range: 59.00-97.00%<br>Mean ± SD: 82.25 ± 8.51%<br>Median: 83.00%<br><br>Absolute humidity:<br>Range: 4.27- 11.63 g/m^3<br>Mean ± SD: 6.69 ± 1.78 g/m^3<br>Median: 6.30 g/m^3	COVID-19 deaths<br><br>Collected from the official website of Health Commission of Hubei Province
Sobral, 2020	Ecological study	World<br>(249 countries)<br><br>Population size not specified<br><br>Number of deaths not specified	December 1, 2019 - March 30, 2020	Weather station data were extracted from the National Oceanic and Atmospheric Administration<br>(NOAA) database. Daily averages were calculated for the different countries.<br><br>Average temperature (Fahrenheit)<br>Maximum temperature (Fahrenheit)	Daily death rates<br><br>Extracted from World Health Organization reports

Author, Year<br>Reference number	Study Design	Study area/<br>Population size:<br><br>Number of cases:	Time period of study	Exposures<br>Exposure data source:<br>Exposure characteristics:	Outcome definition:<br>Outcome source of data:
				Minimum temperature (Fahrenheit)<br><br>No descriptive data included<br><br>Precipitation<br>Average rainfall per country (inches)	
Su, 2020	Ecological study	178 countries/ regions (excluding countries/region without COVID-19 cases and some unmatched countries/region (i.e. Taiwan)<br><br>Statistics of population and cases are based on 6th April 2020.	January 22 - April 6, 2020	Global Surface Summary of the 183 Day (GSOD) via The Integrated Surface Hourly (ISH) dataset (includes global data obtained from the USAF Climatology Center.<br><br>Mean temperature (°Celsius)<br>Relative humidity (%)<br>Precipitation (0.01 inches)	Cumulative Mortality Rate (CMR)<br><br>John Hopkins University dashboard from Center for Systems Science and Engineering
Wu, 2020	Ecological study	Worldwide (166 countries excluding China)<br><br>Population size not given<br><br>23,335 deaths	December - March 27, 2020	Temperature, relative humidity<br><br>The monitoring stations nearest to each country's capital were used.<br>Meteorological data were obtained from the National Oceanic and Atmospheric Administration Center.<br><br>Temperature<br>Range: −5.28 °C to 34.30 °C<br><br>Relative humidity<br>Range: 1.39% to 88.42%	Daily new deaths<br><br>Collected from WHO daily situation reports
Rehman, 2020	Ecological study	Provinces of Pakistan<br><br>No information on population size given	March 10-July 10, 2020	Daily mean humidity and wind, daily and minimum temperature	COVID-19 deaths<br><br>Collected from the government of Pakistan

Author, Year Reference number	Study Design	Study area/ Population size:<br><br>Number of cases:	Time period of study	Exposures<br>Exposure data source:<br>Exposure characteristics:	Outcome definition:<br>Outcome source of data:
		As of July 10, 2020: 5,123 cumulative deaths<br>By area:<br>Sindh: 1,713<br>Punjab: 1,985<br>KPK: 1,074<br>Balochistan: 126<br>Islamabad: 147<br>Gilgit Baltisitan: 36<br>Azad Kashmir: 42		Weather data from Pakistan Meteorological Department (http://www.pmd.gov.pk/en/), https://www.timeanddate.com/weather/pakistan , https://www.accuweather.com, although did not specify which was used.<br><br>Mean humidity<br>Sindh: 61.91%<br>Punjab: 43.41%<br>Khyber Pakhtunkhwa (KPK): 52.24%<br>Balochistan: 32.08%<br>Islamabad: 50.83%<br>Gilgit Baltisitan: 58.19%<br>Azad Kashmir: 57.35%<br><br>Mean wind<br>Sindh: 16.39 Km/h<br>Punjab: 11.95 Km/h<br>KPK: 14.19 Km/h<br>Balochistan: 18.24 Km/h<br>Islamabad: 12.53 Km/h<br>Gilgit Baltisitan: 2.60 Km/h<br>Azad Kashmir: 4.39 Km/h<br><br>Mean Max Temperature (SD)<br>Sindh: 35.23 ^{o}C<br>Punjab: 34.13 ^{o}C<br>KPK: 31.86 ^{o}C<br>Balochistan: 28.37 ^{o}C<br>Islamabad: 31.43 ^{o}C<br>Gilgit Baltisitan: 25.60 ^{o}C<br>Azad Kashmir: 28.11 ^{o}C<br><br>Mean Min Temperature (SD)<br>Sindh: 26.32 ^{o}C<br>Punjab: 22.06 ^{o}C	http://covid.gov.pk/stats/pakistan and Worldometer Coronavirus cases https://www.worldometers.info/coronavirus/country/pakistan/

Author, Year Reference number	Study Design	Study area/ Population size: <br>Number of cases:	Time period of study	Exposures Exposure data source: Exposure characteristics:	Outcome definition: Outcome source of data:
				KPK: 20.63 °C Balochistan: 16.86 °C Islamabad: 20.21 °C Gilgit Baltisitan: 13.50 °C Azad Kashmir: 16.14 °C	
Guo, 2020	Ecological study	415 sites comprising 235 cities from 10 countries and 180 countries <br>Population size not specified <br>119,257 confirmed deaths	January 23-April 13, 2020	Hourly meteorological data (temperature, relative humidity, wind speed) aggregated as daily average meteorological data. If study site had more than one monitoring station, average values of the meteorological factors were weighted by population density. <br>Ground-based monitoring network of the World Meteorological Organization global telecommunications system	COVID-19 mortality <br>Data repository from Johns Hopkins University Center for Systems Science and Engineering (JHU VSSE) The Wind Financial databases (WFD) for detailed information on COVID-19 at city/stae level in Australia, Canada, USA, China, Germany, Italy, Japan, Korea, Norway and Spain
Islam, 2020	Ecological study	Bangladesh <br>Population size not given <br>168 deaths	March 8-April 30, 2020	Night relative humidity (NRH), rainfall, diurnal temperature (TDN), mean temperature (MT), mean relative humidity (MRH), absolute humidity (AH) <br>Bangladesh Meteorological Department (BMD) data from 43 weather stations across the country which were demarcated into eight divisional cites. <br>Diurnal temperature (°C) Mean (SD): 11.3 (1.9) Range: 7.1-16.0 <br>Mean temperature (°C)	COVID-19 death cases <br>Data from Bangladeshi government site

Author, Year<br>Reference number	Study Design	Study area/<br>Population size:<br><br>Number of cases:	Time period of study	Exposures<br>Exposure data source:<br>Exposure characteristics:	Outcome definition:<br>Outcome source of data:
				Mean (SD): 26.6 (1.3)<br>Range: 23.1-28.4<br><br>Mean relative humidity (%)<br>Mean (SD): 64.2 (9.1)<br>Range: 41.6-80.4<br><br>Absolute humidity (%)<br>Mean (SD): 16.5 (2.8)<br>Range: 10.9-21.4<br><br>Rainfall (mm)<br>Mean (SD): 3 (5)<br>Range: 0-20.6	
Jiang and Xu, 2021	Ecological study	Wuhan, China<br><br>No information on population size given<br><br>Daily COVID-19 deaths:<br>Mean: 36.2<br>Min: 0<br>Max: 216	Jan 25 - April 7, 2020	Daily temperature, relative humidity and diurnal temperature range<br><br>Extracted from the Weather Channel (www.weather.com)<br><br>Daily temperature:<br>Mean: 7.4°C, min: 0 °C, Max: 18 °C<br>Relative humidity:<br>Mean: 79.5%, min: 58%, max: 96%<br>Diurnal temperature range:<br>Mean: 7.9 °C, min: 1 °C, max: 16 °C	COVID-19 deaths<br><br>Maintained by the Health Commission of Hubei China
Sun 2020	Ecological study	317 local authority districts (LADs) in England<br><br>3-month COVID-19 mortality rate (deaths per 100,000 persons):<br>Mean: 79.4<br>SD: 36.89	March - May 2020	3-month mean monthly relative humidity and monthly air temperature<br><br>Data set from Met Office HadUK-Grid, Gridded Climate Observations on a 1km grid over the UK	Aggregated three-month England-wide COVID-19 mortality rate<br><br>Spatial patterns of COVID-19 mortality compared to non-COVID-19 mortality

Author, Year Reference number	Study Design	Study area/ Population size: <br>Number of cases:	Time period of study	Exposures Exposure data source: Exposure characteristics:	Outcome definition: Outcome source of data:
				Professional monitoring stations installed in monitoring sites. Monthly relative humidity and monthly air temperature were aggregated from grids to LADs. <br>Average climatic measures of three months (i.e., March, April and May) in 2019 used to represent the climatic measures (i.e., relative humidity and range of air temperature) used in this study. <br>3-month mean relative humidity (%) (R_H): Mean: 76.28 SD: 1.9 <br>3-month mean range of air temperature (°C) (R_AT): Mean: 8.97 SD: 0.84	Office for National Statistics
Tzampoglou and Dimitrios, 2020	Ecologica l study	Worldwide 101 countries, (countries with Human Development Index (HDI) < 0.7 excluded from analysis) <br>No information on population size given <br>Number of deaths not specified	March to May 3, 2020	Monthly average atmospheric temperature (°C), monthly average relative humidity (%), and cumulative precipitation (mm) <br>Collected from the Copernicus Program database, estimated from climate reanalysis ERA-Interim and ERA5 <br>Spatial analysis tool of the ArcGIS software was employed to derive the spatial average of variables across the entire territory of each country. After spatial averaging, temporal average values were computed for the March 2020 to May 2020 period.	Total deaths per million due to COVID-19 <br>Taken from European Commission (EC), OurWorldInData.org, and COVID-19 Government Response Tracker, Blavatnik School of Government

Author, Year Reference number	Study Design	Study area/ Population size: <br>Number of cases:	Time period of study	Exposures Exposure data source: Exposure characteristics:	Outcome definition: Outcome source of data:
Fernández 2021	Ecological study	Worldwide 218 countries <br>No information on population size given <br>Number of deaths not specified	January 21st to May 18th, 2020	Maximum daily temperature and precipitation <br>Downloaded from NASA's Goddard Earth Sciences Data and Information Services Center (GES DISC). <br>Daily precipitation data obtained from Integrated Multi-satellite Retrievals for Global Precipitation Measurement (IMERG) Maximum, minimum, and average, daily temperatures: obtained from the MERRA-2 (a Modern-Era Retrospective analysis for Research and Applications version 2)	Daily confirmed deaths and the total amount of confirmed deaths <br>Based on population-level information (per country), reported by WHO

Table S4. Results of included studies

**Author, Year**<br>**Country**	**Outcome definition:**<br>**Outcome measurement:**	**Confounders/ interaction terms in model:**	**Results**<br>**Analysis methods:**<br>**Lag:**<br>**Summary of results:**
Ma 2020<br>China	COVID-19 deaths<br><br>Collected from the official website of Health Commission of Hubei Province: http://wjw.hubei.gov.cn/	air pollutants, date of the week, time trends	Generalized additive model (GAM) to analyze associations, with a quasi-Poisson link function. Used smoothed spline functions of times to accommodate nonlinear and nonmonotonic patterns between mortality and time.<br><br>Examined single day lag and multiple-day average lag effects (0-5 lag) of weather conditions<br><br>% change of COVID-19 mortality (based on figures 2 and 3 of the text)- no quantitative figures could be obtained.
Sobral 2020<br>Italy	Daily death rates<br><br>Extracted from World Health Organization reports	Population density, dummy month (specific month effects), country's time of exposure to the epidemic (temporal distance, in days, between the first case registered<br>in the territory and the time of study)	Multivariate linear regression<br><br>Lag:<br>No lag effect included<br><br>Results:<br><br>Model 1 (average temperature only):<br>Death: ß= 0.053***<br>***p<0.01<br><br>Model 2 (average temperature, maximum temperature, minimum temperature, precipitation, exposure time):<br>Death:<br>Average temperature: ß= -0.10<br>Maximum temperature: ß= 0.01<br>Minimum temperature: ß= 0.01<br>Precipitation: ß= 0.34<br><br>Model 3 (average temperature, maximum temperature, minimum temperature,

Author, Year<br>Country	Outcome definition:<br>Outcome measurement:	Confounders/ interaction terms in model:	Results<br>Analysis methods:<br>Lag:<br>Summary of results:
			precipitation, exposure time, population density, dummy month):<br>Death:<br>Average temperature: ß= -0.10<br>Maximum temperature: ß= 0.02<br>Minimum temperature: ß= 0.001
Su 2020<br>China	Cumulative Mortality Rate (CMR)<br><br>John Hopkins University dashboard from Center for Systems Science and Engineering	World Development Indicators dataset (World Bank), urban development (% urban population, population growth, population density), GDP per capita, health, infrastructure (railways, passengers carried), poverty (poverty headcount ratio), science and technology (researchers in R&D) , social protection and labor (cover of social insurance programs, unemployment), mean wind speed	Negative binomial regression<br><br>Lag: No consideration of time<br><br>Cumulative mortality rate<br>Mean temperature (^{o}C):<br>IRR= 0.975 (95% CI 0.887-1.071)<br>Relative humidity (%):<br>IRR= 1.025 (95% CI 0.995-1.056)<br>Mean wind speed (.1 knots):<br>IRR= 1.155 (95% CI 0.951-1.403)<br>Precipitation (0.01 inches)<br>IRR= 0.019 (95% CI 0.001-0.377)
Wu 2020<br>China	Daily new deaths<br><br>Cases collected from WHO daily situation reports	Wind speed, median age of national population, Global Health Security Index, Human Development Index, population Density, controlling for countries, date of the week and date of the observation to control time trend and cycle	Log-linear Generalized Additive Model (GAM)<br><br>The lag effects of weather conditions on daily new deaths were considered using single lag days (lag 0, 1, 2, 3). The cumulative effects of average exposure over multiple days were then assessed using additional analyses (lag 01, 02, 03) to control for the possible misalignment of a single lag day exposure.<br><br>Changes in daily new deaths (% change) associated with each 1-unit increase:<br><br>Temperature (^{o}C):<br>ß= -0.65% (95% CI -1.40% to 0.099%)<br>Relative humidity (%)<br>ß= -0.46% (95% CI -0.63% to -0.29%)<br>Controlled for HDI and population density.

Author, Year Country	Outcome definition: Outcome measurement:	Confounders/ interaction terms in model:	Results Analysis methods: Lag: Summary of results:
			Sensitivity analyses:<br>Over 10 days since the first reported case:<br>Temperature (ºC):<br>ß= -1.22% (95% CI -2.00% to -0.45%)<br>Relative humidity (%)<br>ß= -0.51% (95% CI -0.68% to -0.34%)<br><br>Over 100 cumulative cases:<br>Temperature (ºC):<br>ß= -1.25% (95% CI -2.16% to -0.34%)<br>Relative humidity (%)<br>ß= -0.53% (95% CI -0.73% to -0.33%)
Rehman, 2020<br>Pakistan	COVID-19 deaths<br><br>Collected from the government of Pakistan http://covid.gov.pk/stats/pakistan and Worldometer Coronavirus cases https://www.worldometers.info/coronavirus/country/pakistan/	Sun status	Negative Binomial Log Linear Mixed Model<br><br>No lag<br><br>Results: COVID-19 deaths<br><br>Wind<br>Sindh:<br>OR= 1.018 (0.976, 1.062)<br>Punjab:<br>OR= 1.019 (0.985,1.054)<br>KPK:<br>OR= 1.009 (0.979, 1.040)<br>Balochistan:<br>OR= 1.020 (0.996, 1.045)<br>Islamabad:<br>OR= 0.969 (0.932, 1.007)<br>Gilgit Baltisitan:<br>OR= 0.913 (0.741, 1.124)<br>Azad Kashmir:<br>OR= 0.947 (0.871, 1.029)<br><br>Humidity<br>Sindh:<br>OR= 1.001 (0.980, 1.023)<br>Punjab:<br>OR= 1.005 (0.985,1.029)<br>KPK:

Author, Year<br>Country	Outcome definition:<br>Outcome measurement:	Confounders/ interaction terms in model:	Results<br>Analysis methods:<br>Lag:<br>Summary of results:
			OR= 1.002 (0.982, 1.023)<br>Balochistan:<br>OR= 0.981 (0.961, 1.002)<br>Islamabad:<br>OR= 1.017 (0.994, 1.040)<br>Gilgit Baltisitan:<br>OR= 0.998 (0.970, 1.028)<br>Azad Kashmir:<br>OR= 0.986 (0.955, 1.017)<br><br>Temperature daily maximum<br>Sindh:<br>OR= 1.057 (0.957, 1.168)<br>Punjab:<br>OR= 1.076 (0.982,1.16)<br>KPK:<br>OR= 1.100 (0.997, 1.213)<br>Balochistan:<br>OR= 1.085 (0.998, 1.191)<br>Islamabad:<br>OR= 1.100 (1.001, 1.209)<br>Gilgit Baltisitan:<br>OR= 1.026 (0.900, 1.169)<br>Azad Kashmir:<br>OR= 1.108 (0.953, 1.288)<br><br>Temperature daily minimum<br>Sindh:<br>OR= 1.421 (1.307, 1.544)<br>Punjab:<br>OR= 1.194 (1.11,1.296)<br>KPK:<br>OR= 1.065 (0.973, 1.166)<br>Balochistan:<br>OR= 1.002 (0.921, 1.091)<br>Islamabad:<br>OR= 1.197 (1.087, 1.317)<br>Gilgit Baltisitan:<br>OR= 1.105 (0.981, 1.245)<br>Azad Kashmir:<br>OR= 1.1143 (1.004, 1.301)

**Author, Year**<br>**Country**	**Outcome definition:**<br>**Outcome measurement:**	**Confounders/ interaction terms in model:**	**Results**<br>**Analysis methods:**<br>**Lag:**<br>**Summary of results:**
Guo, 2020<br>China	COVID-19 mortality<br><br>Data repository from Johns Hopkins University Center for Systems Science and Engineering (JHU VSSE)<br>The Wind Financial databases (WFD) for detailed information on COVID-19 at city/stae level in Australia, Canada, USA, China, Germany, Italy, Japan, Korea, Norway and Spain	Date of first reported cases, population density, median age, Global Health Security Index (GHSI), latitude, longitude, intervention policies implemented	Negative Binomial Log Linear Mixed Model<br><br>Association of COVID-19 mortality with meteorological factors<br><br>Lag 0-14 days<br>Temperature (Reference=11°C)<br>5°C: RR 1.35 (95% CI: 1.21, 1.51)<br>22°C: RR= 0.51 (95%CI: 0.39, 0.67)<br>Relative humidity (Reference= 71%)<br>59%: RR= 0.98 (95% CI: 0.92-1.05)<br>79%: RR= 0.86 (95% CI: 0.80-0.92)<br>Wind speed (Reference= 3 m/s)<br>2 m/s: RR= 1.31 (95% CI: 1.16, 1.48)<br>4 m/s: RR= 0.76 (95% CI: 0.70, 0.82)<br><br>Lag 0-7<br>Temperature (Reference=11°C)<br>5°C: RR 1.31 (95% CI: 1.20, 1.43)<br>22°C: RR= 0.59 (95%CI: 0.47, 0.74)<br>Relative humidity (Reference= 71%)<br>59%: RR= 0.96 (95% CI: 0.91-1.00)<br>79%: RR= 0.91 (95% CI: 0.86-0.96)<br>Wind speed (Reference= 3 m/s)<br>2 m/s: RR= 1.09 (95% CI: 1.00, 1.19)<br>4 m/s: RR= 0.90 (95% CI: 0.85, 0.95)<br><br>Lag 0<br>Temperature (Reference=11°C)<br>5°C: RR 1.03 (95% CI: 1.00, 1.06)<br>22°C: RR= 0.95 (95%CI: 0.88, 1.03)<br>Relative humidity (Reference= 71%)<br>59%: RR= 1.00 (95% CI: 0.98-1.02)<br>79%: RR= 0.97 (95% CI: 0.96-0.99)<br>Wind speed (Reference= 3 m/s)<br>2 m/s: RR= 0.99 (95% CI: 0.97, 1.01)<br>4 m/s: RR= 1.00 (95% CI: 0.99, 1.01)<br><br>Lag 7<br>Temperature (Reference=11°C)

Author, Year Country	Outcome definition: Outcome measurement:	Confounders/ interaction terms in model:	Results Analysis methods: Lag: Summary of results:
			5°C: RR 1.02 (95% CI: 1.01, 1.04)<br>22°C: RR= 0.95 (95%CI: 0.91, 0.99)<br>Relative humidity (Reference= 71%)<br>59%: RR= 1.00 (95% CI: 0.99-1.00)<br>79%: RR= 1.00 (95% CI: 0.99-1.00)<br>Wind speed (Reference= 3 m/s)<br>2 m/s: RR= 1.03 (95% CI: 1.01, 1.04)<br>4 m/s: RR= 0.98 (95% CI: 0.97, 0.98)<br><br>Lag 14<br>Temperature (Reference=11°C)<br>5°C: RR 1.02 (95% CI: 0.99, 1.06)<br>22°C: RR= 0.92 (95%CI: 0.84, 1.01)<br>Relative humidity (Reference= 71%)<br>59%: RR= 1.00 (95% CI: 0.98-1.02)<br>79%: RR= 1.00 (95% CI: 0.98-1.02)<br>Wind speed (Reference= 3 m/s)<br>2 m/s: RR= 1.03 (95% CI: 1.00, 1.05)<br>4 m/s: RR= 0.98 (95% CI: 0.96, 0.99)<br><br>Shown values of temperature, relative humidity and wind speed correspond to the 25th and 75th percentiles of weather values.
Islam, 2020<br>Bangladesh	COVID-19 death cases<br><br>Data from Bangladeshi government site: https://corona.gov.bd/	None besides the weather parameters shown in results (NRH, TDN, MT, MRH, AH)	Compound Poisson generalized linear model, along with a Monte-Carlo method and random forest model<br><br>Lag: Single and multiple day lags<br><br>Results: no effect numbers (Figures 5 and 6 in the text shows a depiction)
Jiang and Xu, 2021<br>China	COVID-19 deaths<br><br>Maintained by the Health Commission of Hubei China	No further confounders in the analysis model and no government interventions were included because the whole study period was under strict lockdown	Poisson generalized linear model<br><br>Lag: 18 days<br><br>Results<br>Daily temperature<br>ß= − 0.149<br>RR = 0.861 (95% CI: 0.851, 0.872)

Author, Year<br>Country	Outcome definition:<br>Outcome measurement:	Confounders/ interaction terms in model:	Results<br>Analysis methods:<br>Lag:<br>Summary of results:
			Relative humidity<br>ß = − 0.005<br>RR = 0.995 (95% CI: 0.989, 1)<br><br>Diurnal temperature range<br>ß = 0.014<br>RR = 1.014 (95% CI: 1.003, 1.025)
Sun<br>2020<br>England	Aggregated three-month England-wide COVID-19 mortality rate<br><br>Spatial patterns of COVID-19 mortality compared to non-COVID-19 mortality<br>Obtained from Office for National Statistics	First model:<br>sex, ethnicity (percent Asians, percent blacks),<br>percent of households in poverty, unemployment rate, population density, hospital density annual mean $PM_{2.5}$	Variable selection: Lasso technique,<br>Spatial autoregressive model (MESS-SAR),<br>Eigenvector spatial filtering model (RES-ESF)<br><br>Lag: No consideration of time<br><br>Results<br>First model:<br>OLS Model:<br>Humidity: ß= −8.521***<br>Air temperature: ß= −0.795<br><br>MESS-SAR Model:<br>Humidity: ß= −3.715**<br>Air temperature: ß= 1.512<br><br>RE-ESF Model:<br>Humidity: ß= −4.793***<br>Air temperature: ß= 3.852<br><br>Model with selected variables using Lasso technique used humidity but not temperature (results not shown)
Tzampoglou and Dimitrios, 2020<br>Worldwide	Total deaths per million due to COVID-19<br><br>Taken from European Commission (EC), OurWorldInData.org, and COVID-19 Government Response Tracker, Blavatnik School of Government	Cloud cover (CC), population density (PD), median age (MA), stringency index (SI), delay in first case (FC) and stay at-home order measures (SH).<br><br>To include countries that did not impose stay-at-home orders, their SH was time difference between first	Linear Model, Variable Selection: Lasso and forward stepwise<br><br>Lag: No consideration of time<br><br>Results forward stepwise regression:<br>A (95% CIs in parenthesis)<br>Temperature: ß= -108.9 (-307.2, 89.4)

Author, Year<br>Country	Outcome definition:<br>Outcome measurement:	Confounders/ interaction terms in model:	Results<br>Analysis methods:<br>Lag:<br>Summary of results:
		recorded case and end of investigated time period.	Relative humidity: ß= 82.2 (-125.1, 289.5)<br>Precipitation: ß= 13.4 (-258.8, 285.6)<br>confounders: CC, PD, MA, SI, FC, SH<br>B<br>Temperature: ß= -88.9 (-259.2, 81.5)<br>Relative humidity: ß= 79.1 (-126.5, 284.8)<br>Precipitation: ß= -17.9 (-239.6. 203.8)<br>Confounders: PD, MA, SI, FC, SH<br>C<br>Temperature: ß= -86.7 (-286-3, 112.9)<br>Relative humidity: ß= 34.8 (-168.9, 238.5)<br>Precipitation: ß= 11.3 (-264.7, 287.3)<br>Confounders: CC, PD, MA, FC, SH<br>D<br>Temperature: ß= -89.7 (-262.3, 82.9)<br>Relative humidity: ß= 34.9 (-167.6, 237.3)<br>Precipitation: ß= 16.3 (-205.0, 237.6)<br>Confounders: PD, MA, FC, SH<br>F<br>Temperature: ß= -60.6 (-222.9, 101.7)<br>Relative humidity: ß= 62.7 (-131.8, 257.2)<br>Precipitation: ß= -14.8 (-227.1. 197.5)<br>Confounders: MA, FC, SH<br>G<br>Temperature: ß= -86.8 (-253.8, 80.2)<br>Relative humidity: ß= 46.1 (-86.6, 178.8)<br>Confounders: PD, MA, FC, SH<br>H<br>Temperature: ß= -103.9 (-275.0, 67.2)<br>Relative humidity: ß= 34.7 (-168.0, 237.5)<br>Precipitation: ß= 28.1 (-192.7, 248.8)<br>Confounders: PD, MA, FC,<br>I<br>Temperature: ß= -99.1 (-265.1, 66.9)<br>Relative humidity: ß= 54.2 (-78.0, 186.4)<br>Confounders: PD, MA, FC<br><br>Temperature: ß= -90.9 (-258.6, 76.8)<br>Relative humidity: ß= 40.6 (-92.5, 173.7)<br>Confounders: PD, MA, SH

Author, Year<br>Country	Outcome definition:<br>Outcome measurement:	Confounders/ interaction terms in model:	Results<br>Analysis methods:<br>Lag:<br>Summary of results:
			Results of Lasso analysis were not shown, but authors stated that they agreed with forward stepwise analysis.
Fernández 2021<br>Worldwide	Daily confirmed deaths and the total amount of confirmed deaths<br><br>Based on population-level information (per country), reported by WHO	National Biodiversity Index (NBI), Population density, days since last case, days since first case reported in country, country income level, government intervention level	Generalized linear mixed models<br><br>Lag: 14 days<br><br>Results of Bayesian spatio-temporal regression analysis:<br>All countries<br>Precipitation: ß= 0.000 (95% CI: -0.002, 0.001)<br>Maximum temperature: ß= -0.003 (95% CI: -0.010, 0.005)<br><br>Excluding top five countries with largest area (Russia, Canada, China, USA and Brazil)<br>Precipitation: ß= -0.001 (95% CI: -0.002, 0.002)<br>Maximum temperature: ß= 0.074 (95% CI: -0.025, 0.205)
